# IL-10 Restores MHC Class I Expression and Interferes With Immunity in Papillary Thyroid Cancer With Hashimoto Thyroiditis

**DOI:** 10.1210/endocr/bqaa062

**Published:** 2020-04-29

**Authors:** Zhong-Wu Lu, Jia-Qian Hu, Wan-Ling Liu, Duo Wen, Wen-Jun Wei, Yu-Long Wang, Yu Wang, Tian Liao, Qing-Hai Ji

**Affiliations:** 1 Department of Head and Neck Surgery, Fudan University Shanghai Cancer Center, Shanghai, China; 2 Department of Oncology, Shanghai Medical College, Fudan University, Shanghai, China

**Keywords:** papillary thyroid cancer, Hashimoto thyroiditis, tumor immunity, MHC classⅠmolecule, interleukin-10

## Abstract

The incidence of papillary thyroid cancer (PTC) with concomitant Hashimoto thyroiditis (HT) is increasing. Interleukin (IL)-10 is a cytokine previously reported to be elevated in this condition. Evidence from multiple human malignancies showed IL-10 participated in tumor immunity and exhibited therapeutic potential. The aim of this study is to investigate whether IL-10 interferes with tumor immunity in PTC with concomitant HT. Expression of IL-10 and major histocompatibility complex (MHC) class Ⅰ were compared with PTC tissues with or without concomitant HT. PTC cell lines K1 and TPC-1 were stimulated with IL-10 and analyzed for MHC class Ⅰ expression afterward. T-cell activation, production of IL-2 and interferon (IFN)-γ and programmed death-1 (PD-1) expression were assessed by coculture of donor peripheral blood lymphocytes (PBLs) with IL-10-pretreated PTC cells. Programmed death-ligand 1 (PD-L1) expression was measured in PTC tissues and IL-10-pretreated cells of K1 and TPC-1. Increased levels of IL-10 and MHC class Ⅰ were observed in PTC with concomitant HT. IL-10 stimulation increased MHC class Ⅰ expression of PTC cells in vitro. Coculture of PBLs with IL-10-pretreated PTC cells enhanced T-cell activation (% cluster of differentiation [CD]25^+^ of CD3^+^T cells) and increased IL-2 production along with decreased IFN-γ secretion and PD-1 expression. Reduced PD-L1 expression was seen in PTC + HT tissue samples and IL-10-stimulated PTC cell lines. Elevated IL-10 expression in PTC with concomitant HT restores MHC class Ⅰ expression and interferes with tumor immunity. The potential mechanism of IL-10 in tumor immunity needs further investigation.

Thyroid cancer (TC) is the most common malignancy of the endocrine system. Due to overdiagnosis and routine ultrasound screening, TC demonstrates a steadily growing incidence over the past few decades (1). Papillary thyroid cancer (PTC) is the predominant histotype of TC and is frequently found to be associated with Hashimoto thyroiditis (HT) in postoperative pathology reports. The number of patients who suffer from both PTC and HT has increased significantly since Dailey et al (1955) first reported the concomitance of the 2 diseases in 1955 (2-6).

Vast numbers of studies have tried to elucidate the possible relationship between PTC and HT. Despite the dedication devoted to this topic, no consensus could be reached to date. Notably, the majority of those studies, including 1 completed by our department, considers HT as a protective factor for PTC, which results in a better prognosis (3, 7-9). However, the underlying mechanism remains unknown.

HT typically presents with diffused infiltrated lymphocytes, which are recruited by cytokine secretion during immune response. We focused on 1 specific cytokine, interleukin (IL)-10, whose expression is found to be elevated in PTC with concomitant HT (10). More importantly, evidence showed that IL-10 effectively induced activation of CD8^+^ T cells in several mouse tumor models, suggesting its therapeutic potential in cancer immunotherapy (11, 12).

The antitumor cluster of differentiation (CD) 8^+^ T cells attack major histocompatibility complex (MHC) class-positive cancer cells. However, human malignancies, including PTC, tend to downregulate MHC class Ⅰ expression as one of their immune escape strategies (13-15). Therefore, recovery of MHC class I expression is critical to effective immunotherapy.

The aim of this study was to evaluate the role of IL-10 in patients with PTC with concomitant HT, analyzing its potential function from the view of tumor immunity through in vitro experiments.

## Material and Methods

### Patients and tissue samples

This study recruited 140 surgically treated patients with PTC with or without HT during April 2014 and January 2016 at the department of Head and Neck Surgery, Fudan University Shanghai Cancer Center. Diagnosis of PTC and HT was based on postoperative pathology reports. Patients with immunodeficiency were excluded from the study. None of the patients had a previous history of any kind of treatment for their thyroid condition before surgery.

Fresh frozen samples were obtained from 69 patients during surgery. Formalin-fixed and paraffin-embedded tissue sections of the rest 71 participants were acquired from the hospital tissue bank. Clinical data (sex; age; tumor size; extrathyroidal invasion; lymph node metastasis; multifocality; and tumor, node, and metastasis [TNM] classification) were collected. TNM classification was determined according to the eighth edition of American Joint Committee on Cancer and International Union Against Cancer TNM staging criteria. Each patient signed informed consent for the use of their tissue samples before research. This study acquired Institutional Review Board approval from Fudan University Shanghai Cancer Center.

### Cell lines and culture

Immortalized nonmalignant human thyroid cell line Nthy-ori 3-1 and 2 PTC cell lines (K1 and TPC-1) were used in this study. Nthy-ori 3-1 was purchased from Sigma-Aldrich, Inc, and cells of K1 and TPC-1 were purchased from University of Colorado Cancer Center Cell Bank. Cells were cultured in Roswell Park Memorial Institute 1640 medium containing 10% fetal bovine serum (Invitrogen, Carlsbad, CA, USA) at 37℃ with 5% carbon dioxide in proper humidity.

### Ribonucleic acid extraction, reverse transcription, and quantitative real-time polymerase chain reaction

Total ribonucleic acid of cells and tissue samples was extracted with TRIzol Reagent (Invitrogen, Inc.) and complementary deoxyribonucleic acid synthesis was performed by PrimeScript RT Reagent Kit (Takara, Dalian, China). Using SYBR Green Premix Ex Taq II (Takara, Dalian, China), quantitative real-time polymerase chain reaction (qPCR) was then conducted in triplicate. IL-10 (forward 5ʹ-3ʹ: TCTCCGAGATGCCTTCAGCAGA; reverse 5ʹ-3ʹ: TCAGACAAGGCTTGGCAACCCA), HLA (human leukocyte antigen)-A (forward 5ʹ-3ʹ: GTGGCCTCATGGTCAGAGAT; reverse 5ʹ-3ʹ: GCAGTTGAGAGCCTACCTGG), HLA-B (forward 5ʹ-3ʹ: GTGATCTCCGCAGGGTAGAA; reverse 5ʹ-3ʹ: TCCGCAGATACCTGGAGAAC), HLA-C (forward 5ʹ-3ʹ: TGATCTCCGCAGGGTAGAAG; reverse 5ʹ-3ʹ: CAGATACCTGGAGAACGGGA), and PD-L1 (forward 5ʹ-3ʹ: CCATACAGCTGAATTGGTCATC; reverse 5ʹ-3ʹ: CAGAATTACCAAGTGAGTCCTTTCA) were tested and β-actin (forward 5ʹ-3ʹ: CACCATTGGCAATGAGCGGTTC; reverse 5ʹ-3ʹ: AGGTCTTTGCGGATGTCCACGT) served as an internal control for messenger ribonucleic acid assays. Results were analyzed according to comparative cycle threshold values (2^-ΔΔCt^).

### Immunohistochemistry

Formalin-fixed and paraffin-embedded tissue sections were deparaffinized in xylene and rehydrated with ethanol. Blocking of endogenous peroxidase activity was conducted using 3% hydrogen peroxide. After heat-induced antigen retrieval (0.01 mol/L citrate, pH 6.0), 5% bovine serum albumin was used to block nonspecific protein-protein interactions. Sections were then incubated overnight with primary antibodies against IL-10 (20850-1-AP, Proteintech), HLA class I ABC (ab70328, Abcam) and PD-L1 (13684T, Cell Signaling Technology). Secondary antibody staining and antigen detection were performed using a horseradish peroxidase–conjugated rabbit or mouse immunohistochemistry (IHC) kit (KIHC-1, Proteintech). Sections were counterstained with hematoxylin. Images were obtained through an Olympus IX71 inverted microscope with a DP2-BSW Olympus image acquisition software system (Olympus, Japan).

### Isolation of peripheral blood lymphocytes

Peripheral blood of healthy donors was drawn through routine venipuncture. Peripheral blood lymphocytes (PBLs) were isolated using human lymphocyte separation medium (Dakewe Biotech Co, Ltd) through differential density gradient centrifugation. Cells were plated in U-shaped bottom 96-well cell culture plates (2 × 10^5^ cells/well) using the Roswell Park Memorial Institute 1640 medium containing 10% fetal bovine serum (Invitrogen, Carlsbad, CA, USA) at 37℃. Antibodies against CD3 (16-0037-85, eBioscience) and CD28 (16-0289-85, eBioscience) were added into the media at a concentration of 2 μg/mL. After 72 hours, PBLs were dyed with fluorescently conjugated antibodies against CD3 (300 308, BioLegend), CD8 (300 906, BioLegend), and CD25 (302 610, BioLegend) and then sorted by flow cytometer (MoFlo XDP, Beckman Coulter, Inc). Activated T cells (CD3^+^CD8^+^CD25^+^) were collected.

### Pretreatment of PTC cell lines with IL-10

Recombinant human IL-10, 0.1 μg/μL, (200-10, Peprotech) was added into the culture media of K1 and TPC-1 cells for 24 hours. The cells were then collected and washed by phosphate-buffered saline to thoroughly remove the residue of IL-10.

### Coculture system of activated T cells (CD3^+^CD8^+^CD25^+^) and PTC cell lines

CD3^+^CD8^+^CD25^+^ T cells (effector cells, E) were seeded in flat bottom 24-well cell culture plates (1 × 10^5^ cells/well) along with PTC cells (targeted cells, T) at an effector cell:targeted cell (E:T) ratio of 10:1 or 30:1 for 24 hours before further experimentation.

### Flow cytometry

Cells were collected and dyed with fluorescently conjugated antibodies against MHC class I ABC (ab70328, Abcam), CD3 (300308, BioLegend), CD25 (302610, BioLegend) and PD-1 (329918, BioLegend). Flow cytometry was performed using a Cytomics FC 500 cytometer (Beckman Coulter, Inc). For the detection of MHC class I expression in PTC cell lines, cells were also stained with fluorescein isothiocyanate-labeled goat anti-mouse secondary antibody (555988, BD Pharmingen). Results were analyzed using FlowJo software (Tree Star).

### Enzyme-linked immunosorbent assay

The supernatant fluid of coculture systems was analyzed for IL-2 and IFN-γ concentration using the precoated human IL-2 enzyme-linked immunosorbent assay (ELISA) kit (12-1020-096, Dakewe Biotech Co, Ltd) and human IFN-γ ELISA kit (12-1000-096, Dakewe Biotech Co, Ltd). Briefly, samples and prediluted standards were added to precoated wells. The detection antibody was then added, followed by incubation at room temperature for 1 (IL-2) or 2 (IFN-γ) hours. Horseradish peroxidase conjugate was added and incubated at room temperature for 20 minutes. To develop the plate, 3,3'5,5'-tetramethyl benzidine dihydrochloride was used. Once the stop solution was added, the absorbance of each well was read at 450 nm by Synergy H4 Hybrid microplate reader (BioTek).

### Western blot analysis

Cell lysates were obtained from 1×10^6^ cultured cells with a mixture of radioimmunoprecipitation assay protein extraction reagent, protease inhibitor, and phosphatase inhibitor (Roche, CA, USA). Thyroid tissue samples were treated with T-PE Tissue Protein Extraction Reagent (Thermo Scientific) and lysed by Vibra-Cell Ultrasonic Liquid Processors (Sonics & Materials, Inc). Protein concentration was measured using a bicinchoninic acid assay. Equal amounts of total protein lysate were separated by 10% sodium dodecyl sulfate–polyacrylamide gel electrophoresis and transferred onto polyvinylidene difluoride membranes. The membranes were then blocked in 5% nonfat milk and probed with primary antibodies against MHC class I (1:1000, Abcam), PD-L1 (1:1000, Cell Signaling Technology), and glyceraldehyde 3-phosphate dehydrogenase (1:5000, Abcam) at 4℃ overnight. After incubation in a solution of goat–anti-rabbit or anti-mouse IgG (1:5000 for both; Jackson ImmunoResearch Laboratories) at room temperature for 1 hour, the membranes were treated with enhanced chemiluminescence reagents (Thermo Fisher Scientific). Bands were detected with Alpha Imager (Alpha Innotech, San Leandro, CA, USA).

### Statistical analysis

All data in the study are shown as mean ± standard deviation or standard error of the mean as indicated. Independent *t* tests were used for continuous variables and the Pearson χ2 tests were used for categorical variables. *P < 0*.05 was considered to indicate a statistically significant difference. Statistical tests were performed using GraphPad Prism 5.01 software (GraphPad Software, Inc) and IBM SPSS 22.0 (Armonk, NY, USA). Graphs and figures were produced using GraphPad and Adobe Photoshop (Adobe Systems Inc).

## Results

### Patient characteristics

Samples from 140 patients with PTC were used in this study. Of these patients, 110 (78.6%) were female. A total of 117 (83.6%) patients were aged <55 years. About half (n=76, 54.3%) of the study cohort had tumors that were less than 1 cm. Multifocal lesions were detected in 44 (31.4%) individuals. Extrathyroidal invasion only occurred in 18 (12.9%) patients. Lymph node metastasis was positive in 75 (53.6%) patients, while no distant metastasis was identified in the whole cohort. A total of 137 (95.1%) of the patients were classified as stage I or II according to the latest eighth edition of the American Joint Committee on Cancer and International Union Against Cancer TNM staging system.

Based on postoperative pathology reports, 51 (36.4%) patients had HT in addition to PTC. This subgroup consisted of more female patients than the PTC group (92.2% vs 70.8%; *P = 0*.003). The age, multifocal lesions, extrathyroidal invasion, lymph node metastasis, and TNM classification between patients with PTC with or without HT showed no difference. Notably, tumor size in these 2 groups varied significantly. Patients with PTC with HT had bigger tumors than those without concomitant HT (1.4 ± 1.0 vs 1.1 ± 0.7; *P = 0*.031). When patients were further divided according to the size of tumor (tumor size ≤ 1 and > 1 cm), data revealed more microcarcinoma in the PTC group than in the PTC + HT group (61.8% vs 41.2%; *P = 0*.018). Clinical characteristics of the 140 patients with PTC in this study are listed in Table 1.

**Table 1.** Clinical Characteristics of All Patients in this Study

	PTC	PTC + HT	
Clinicopathologic Parameters	N (%)	N (%)	*P* Value
**Gender**			0.003**
Male	26 (29.2)	4 (7.8)	
Female	63 (70.8)	47 (92.2)	
**Age (years)**			0.159
Mean	42.6 ± 12.4	39.5 ± 13.1	
< 55	73 (82.0)	44 (86.3)	0.513
≥ 55	16 (18.0)	7 (13.7)	
**Tumor size (cm)**			
Mean	1.1 ± 0.7	1.4 ± 1.0	0.031*
≤ 1	55 (61.8)	21 (41.2)	0.018*
> 1	34 (38.2)	30 (58.8)	
**Multifocal lesions**			0.060
Positive	23 (25.8)	21 (41.2)	
Negative	66 (74.2)	30 (58.8)	
**Extrathyroidal invasion**			0.200
Positive	9 (10.1)	9 (17.6)	
Negative	80 (89.9)	42 (82.4)	
**Lymph node metastasis**			0.346
Positive	45 (50.6)	30 (58.8)	
Negative	44 (49.4)	21 (41.2)	
**TNM stage 8** ^**th**^a			0.271
I, II	88 (98.9)	49 (96.1)	
III, IV	1 (1.1)	2 (3.9)	

Patients in this study: n=140.*P < 0.05; ^**^P < 0.01.

Abbreviations: HT, Hashimoto thyroiditis; PTC, papillary thyroid carcinoma; TNM, tumor, node, metastasis.

aThe 8th edition of American Joint Committee on Cancer and International Union Against Cancer TNM Staging System.

### HT is related to higher IL-10 expression in PTC

**Figure 1.**
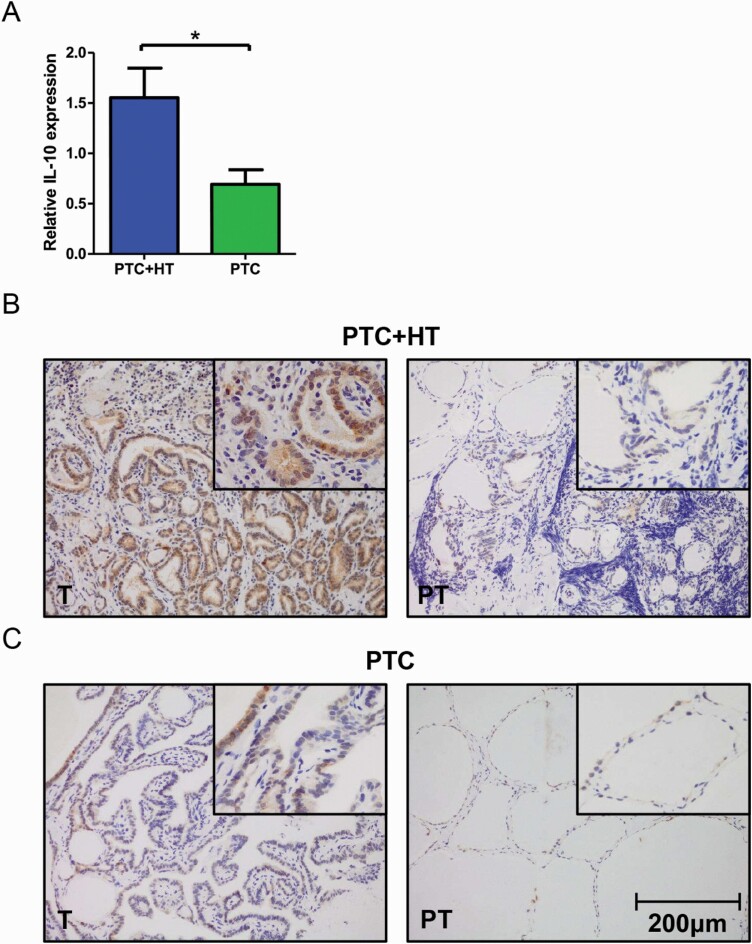
Increased IL-10 expression in PTC + HT tissues. (**A**) qPCR result of relative IL-10 expression in tumor tissues from patients with PTC with (n = 37) or without HT (n = 32). qPCR tests were repeated 3 times (*P < 0.05). (**B-C**) Representative pictures of IHC staining of IL-10 in PTC + HT tumor tissue, PTC + HT adjacent paratumor tissue (**B**) and PTC tumor tissue and PTC adjacent paratumor tissue (**C**) (original magnification, 200×; the right upper quadrant, 400×). HT, Hashimoto thyroiditis; IL, interleukin; PT, paratumor; PTC, papillary thyroid cancer; qPCR, real-time polymerase chain reaction; T, tumorous.

The effect of HT on IL-10 expression in PTC was measured by qPCR in fresh frozen tissue samples of 69 patients with PTC. A total of 37 (53.6%) of these patients had HT in addition to PTC, and they presented with a higher expression of IL-10 according to the test result (*P < 0*.05, Fig. 1A). Comparison of clinical data was carried out between groups with high IL-10 expression and groups with low IL-10 expression. A total number of 39 (56.5%) patients was attributed to the IL-10 high-expression group. No remarkable distinction was found in sex, age, multifocal lesions, extrathyroidal invasion, lymph node metastasis, and TNM classification between the 2 groups. Notably, patients in the IL-10 high-expression group were more vulnerable to tumors > 1cm (66.7% vs 40%; *P = 0*.027). The clinical information of 69 patients with PTC in the qPCR cohort is listed in Table 2.

**Table 2.** Clinical characteristics of patients with PTC in the qPCR cohort

	IL-10 Low	IL-10 High	
Clinicopathologic Parameters	N (%)	N (%)	*P* Value
**Gender**			0.616
Male	6 (20.0)	6 (15.4)	
Female	24 (80.0)	33 (84.6)	
**Age (years)**			
Mean	38.6 ± 14.2	40.8 ± 13.4	0.517
< 55	28 (93.3)	34 (87.2)	0.401
≥ 55	2 (6.6)	5 (12.8)	
**Tumor size (cm)**			
Mean	1.2 ± 0.8	1.3 ± 0.9	0.479
≤ 1	18 (60.0)	13	0.027*
> 1	12 (40.0)	26	
**Multifocal lesions**			0.063
Positive	6 (20.0)	16	
Negative	24 (80.0)	23	
**Extrathyroidal invasion**			0.435
Positive	4 (13.3)	8	
Negative	26 (86.7)	31	
**Lymph node metastasis**		0.512	
Positive	13 (43.3)	20	
Negative	17 (56.7)	19	
**TNM stage 8** ^**th**^a			0.208
I, II	30 (100.0)	37	
III, IV	0 (0.0)	2	

Patients in this cohort: n=69.*P < 0.05. Abbreviations: IL-10: Interleukin-10; PTC: papillary thyroid carcinoma; qPCR, real-time polymerase chain reaction; TNM, tumor, node, metastasis.

aThe 8th edition of American Joint Committee on Cancer and International Union Against Cancer TNM Staging System.

Paraffin-embedded sections of 71 patients with PTC were stained with IL-10 antibody. Results of IHC staining were compared between tumorous (T) and adjacent paratumor (PT) tissues from patients with PTC with and/or without HT. For each patient group, the tumor tissue exhibited a higher expression of IL-10 than adjacent paratumor tissue. More extensive and higher intensity of IL-10 staining can be seen on tumor samples with PTC + HT compared with PTC (Fig. 1B-C). However, the difference between adjacent paratumor tissues from the 2 patient groups was not clear. Additionally, diffuse lymphocyte infiltration, which was caused by autoimmune response, can be observed on both T and PT sections from PTC + HT.

### HT is related to higher MHC class I expression in PTC

**Figure 2.**
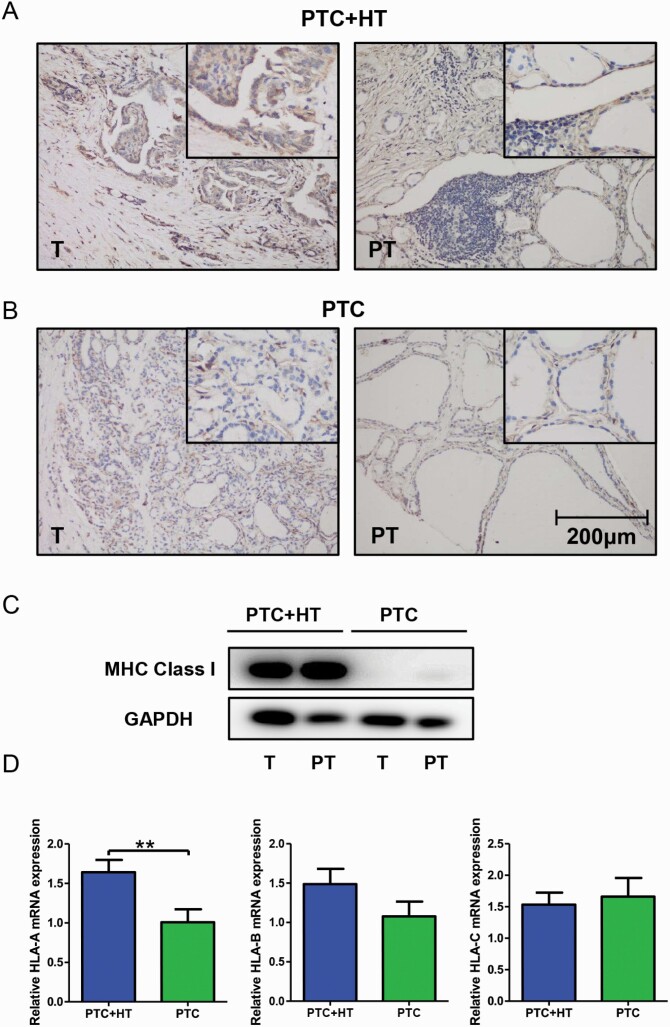
Increased MHC class I expression in PTC + HT tissues. (**A-B**) Representative pictures of IHC staining of MHC class I molecules in PTC + HT tumor tissue. PTC + HT adjacent paratumor tissue (**A**) and PTC tumor tissue and PTC adjacent paratumor tissue (**B**) (original magnification, 200×; the right upper quadrant, 400×). (**C**) MHC class I expression in tissue samples of patients with PTC and patients with PTC + HT measured by Western blot; test was repeated 3 times. (**D**) qPCR results of relative HLA-A, HLA-B, and HLA-C expression between tumor tissues from patients with PTC with (n = 37) or without HT (n = 32); tests were conducted in triplicate (**P < 0.01). GAPDH, glyceraldehyde 3-phosphate dehydrogenase; HT, Hashimoto thyroiditis; HLA, human leukocyte antigen; MHC, major histocompatibility complex; mRNA, messenger ribonucleic acid; PT, paratumor; PTC, papillary thyroid cancer; qPCR, real-time polymerase chain reaction; T, tumorous.

IHC staining and Western blot analysis were performed to evaluate the effect of concomitant HT on MHC class I expression in PTC. As shown in Figure 2A and 2B, a more abundant expression of MHC class I was perceived in tissue sections from PTC + HT compared with that from PTC, in both tumor and adjacent paratumor tissues. Among all 4 pictures, PTC + HT tumor tissue possessed the highest expression of MHC class I molecules. The Western blot result in Figure 2C demonstrated a remarkably increased MHC class I expression in proteins from PTC + HT, which was consistent with the findings of IHC.

Tissue samples were then further examined for HLA-A, HLA-B, and HLA-C expression by qPCR. Data were analyzed between patients with PTC with and without HT. We detected a significantly higher expression of HLA-A in tumors from PTC + HT (*P < 0*.01, Fig. 2D). As for the HLA-B and HLA-C gene, no difference in expression was discovered between the 2 groups.

### IL-10 induces MHC class I expression in PTC cells in vitro

We have demonstrated that concomitant HT was related with increased expression of IL-10 and MHC class I, respectively. To evaluate the correlation between IL-10 and MHC class I expression, normal human thyroid cell line Nthy-ori 3-1 and PTC cell lines K1 and TPC-1 were used in this study.

**Figure 3.**
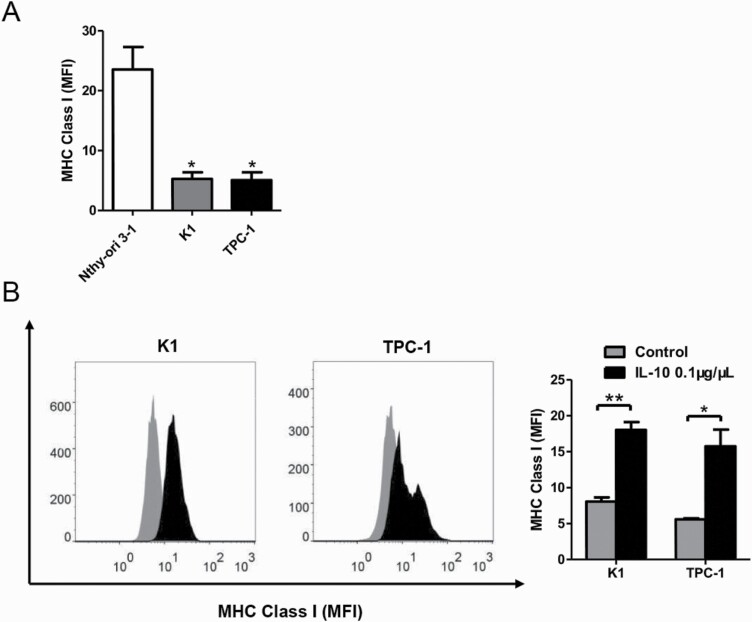
IL-10 induces MHC class I expression in PTC cell lines. (**A**) MHC class I expression measured by flow cytometry in normal human thyroid cell line Nthy-ori 3-1, PTC cell lines K1 and TPC-1 (*P < 0.05). (**B**) The effect of in vitro stimulation with 0.1 μg/μL of IL-10 for 24 hours on MHC class I expression of PTC cell lines (K1 and TPC-1) measured by flow cytometry (*P < 0.05, **P < 0.01); tests were repeated 3 times. IL, interleukin; MFI, fluorescence intensity; MHC, major histocompatibility complex; PTC, papillary thyroid cancer; T, tumorous.

We first examined the baseline expression of MHC class I molecules in each cell line using flow cytometry. Compared with Nthy-ori 3-1, cells of K1 and TPC-1 both presented reduced mean fluorescence intensity of MHC class I (*P < 0*.05, Fig. 3A). The PTC cells were then stimulated with 0.1 μg/μL recombinant human IL-10 for 24 hours. Flow cytometry results showed significantly increased expression of MHC class I molecules in both cell lines after IL-10 stimulation (*P < 0*.01 and *P < 0*.05, Fig. 3B).

### IL-10 enhances T-cell activation in vitro

**Figure 4.**
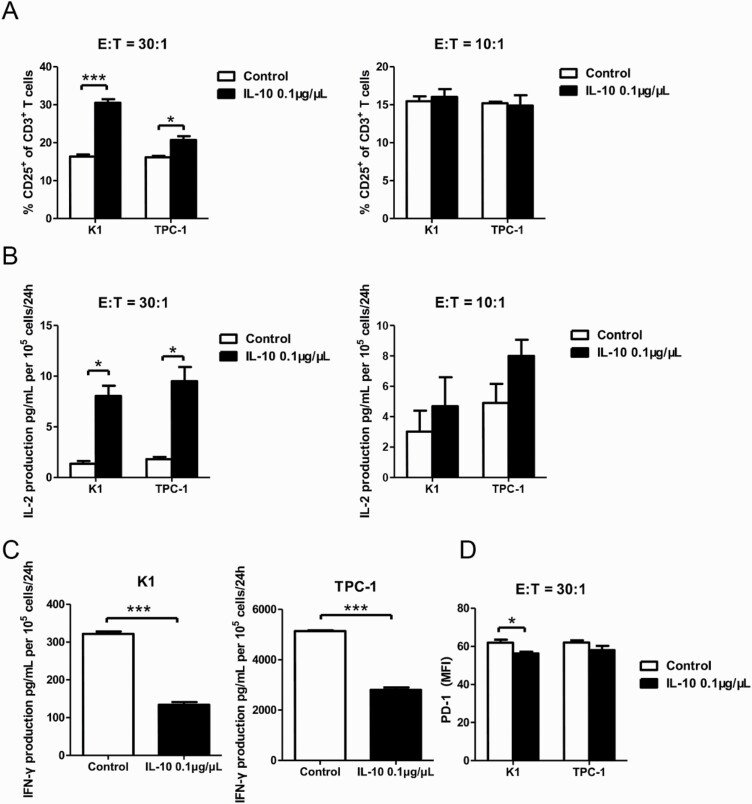
IL-10 promotes T-cell activation in cocultures of CD3^+^CD8^+^CD25^+^ T cells and PTC cell lines. (**A**) Flow cytometry results of the CD25^+^ fraction of CD3^+^ T cells in coculture systems containing activated CD3^+^CD8^+^CD25^+^ T lymphocytes (effector cells, E) and pretreated PTC cells (K-1 and TPC-1 treated with 0.1 μg/μL of IL-10 for 24 hours; target cells, T) at various E:T ratios (30:1 and 10:1) (*P < 0.05, ***P < 0.001). (**B**) IL-2 production in coculture systems (details described above) measured by ELISA (*P < 0.05). (**C**) IFN-γ production in coculture systems of CD3^+^CD8^+^CD25^+^T lymphocytes and IL-10-stimulated PTC cells (K-1 and TPC-1) at a 30:1 ratio (***P < 0.001). (**D**) Mean fluorescence intensity (MFI) of PD-1 expression in coculture systems of activated CD3^+^CD8^+^CD25^+^ T lymphocytes and IL-10 pretreated PTC cells at a 30:1 ratio (*P < 0.05). All tests were conducted in triplicate. ELISA, enzyme-linked immunosorbent assay; H, hours; IFN, interferon; IL, interleukin; PD-1, programmed death-1; PTC, papillary thyroid cancer.

CD3^+^CD8^+^CD25^+^T cells isolated from heathy donors were cocultured with IL-10-pretreated cells of K1 and TPC-1 for 24 hours. The effector cells (E, T cells) and targeted cells (T, PTC cells) were planted at an E:T ratio of 30:1 or 10:1. The proportion of CD25^+^T cells of all CD3^+^T cells was determined by flow cytometry. As shown in Figure 4A, a higher proportion of activated CD3^+^CD8^+^CD25^+^T cells appeared in both cell lines after IL-10 administration when the E:T ratio was set at 30:1 (*P < 0*.001 and *P < 0*.05). However, when the ratio was lowered to 10:1, no noticeable distinction could be observed.

ELISA was used to measure IL-2 secretion of activated T cells from cocultures. Results showed IL-10 successfully increased the amount of IL-2 production in both K1 and TPC-1 cocultures. Significance appeared at the 30:1 ratio (*P < 0*.05, Fig. 4B), while the E:T ratio of 10:1 failed to show a difference. IFN-γ secretion in those cocultures at an E:T ratio of 30:1 was also evaluated by ELISA. Data showed T cells cocultured with TPC-1 cells possessed greater ability of IFN-γ secretion than those cocultured with K1 cells, regardless of IL-10 stimulation. With the pretreatment of IL-10, IFN-γ secretion of T cells dropped dramatically in cocultures of both PTC cell lines (*P < 0*.001, Fig. 4C).

PD-1 expression on activated T cells in cocultures was measured by flow cytometry at an E:T ratio of 30:1. Compared with control groups, the extra treatment of IL-10 in K1 cocultures effectively lowered the expression of PD-1 on T cells (*P < 0*.05, Fig. 4D). Unfortunately, the expression difference in TPC-1 cocultures was not conclusive.

### IL-10 regulates T-cell activation through PD-1/PD-L1 pathway in vitro

**Figure 5.**
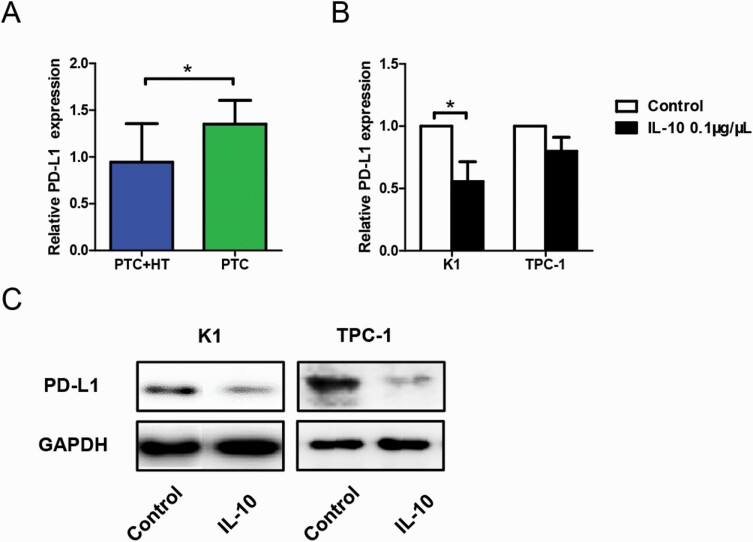
Effects of concurrent HT and in vitro IL-10 stimulation on PD-L1 expression in PTC. (**A**) qPCR result of relative PD-L1 expression in tumor tissues from patients with PTC with (n = 37) or without HT (n = 32) (*P < 0.05). (**B-C**) Effect of in vitro treatment of IL-10 (0.1 μg/μL) for 24 hours on PD-L1 expression of PTC cell lines (K1 and TPC-1) measured by qPCR (**B**) and Western blot (**C**) (*P < 0.05). All tests were conducted in triplicate. GAPDH, glyceraldehyde 3-phosphate dehydrogenase; HT, Hashimoto thyroiditis; IL, interleukin; PD-L1, programmed death-ligand 1; qPCR, real-time polymerase chain reaction; PTC, papillary thyroid cancer.

Our previous results showed PD-1 expression was reduced in cocultures of activated CD3^+^CD8^+^CD25^+^T cells and IL-10 pretreated K1 cells at an E:T ratio of 30:1. As the other half of the co-stimulatory molecules, expression of PD-L1 was then tested by qPCR. Data analysis revealed reduced PD-L1 expression in tumor tissues from patients with PTC + HT (*P < 0*.05, Fig. 5A), indicating the downregulation of T-cell apoptosis.

Cells of K1 and TPC-1 were then stimulated with 0.1 μg/μL recombinant human IL-10 for 24 hours and measured for their expression of PD-L1 using qPCR and Western blot analysis. The qPCR result in Figure 5B showed downregulated PD-L1 expression on K1 cells after IL-10 stimulation (*P < 0*.05). However, the change of PD-L1 expression on TPC-1 cells was not quite clear. The Western blot results of both K1 and TPC-1 cells revealed reduced PD-L1 expression under the influence of IL-10 administration (Fig. 5C).

## Discussion

The relationship between HT and PTC has always been intriguing (4). According to extensive research in this field, a diagnosis of PTC with concomitant HT normally had a better prognosis (7-9, 16). A former study conducted in our institute pointed out that the presence of HT, though a risk factor for PTC diagnosis, served as a protective factor for central compartment lymph node metastasis (3). However, contradictory opinions also occur where HT may act as a risk factor or harbor no potential contribution to a PTC prognosis at all (5, 6). In our study, clinical statistics showed patients with PTC with concomitant HT had bigger tumors than patients with PTC, while the TNM classification of the 2 groups revealed no significant difference. Tumor size related directly to T classification and was generally considered a risk factor in PTC. Therefore, the inconsistency between advanced T classification and the overall TNM classification in the PTC + HT group may suggest the protective role of HT to some degree.

Development of HT involves a series of complicated immune responses that ultimately lead to cytokine release, lymphocyte infiltration, and cell destruction in the thyroid gland. Of special interest is IL-10, whose polymorphism was associated with a 3-fold increased risk of HT (17). Notably, IL-10 has also been reported to contribute to tumorigenesis in multiple human malignancies, including colorectal and cervical carcinoma (18, 19). Stanciu et al (2015) reported that serum IL-10 levels were substantially higher in patients with PTC + HT with persistent/recurrent disease compared with patients with PTC (with or without recurrence) (10). Unfortunately, we were unable to conduct the similar comparison due to our insufficient survival information of the patient cohort. Nevertheless, our data confirmed an elevated IL-10 expression in tumor tissue from patients with PTC with concomitant HT both by qPCR and IHC staining. When patients were classified by their IL-10 expression, differences appeared in tumor size. The IL-10 low-expression group harbored more microcarcinoma compared with the IL-10 high-expression group. This result, combined with our previous finding that patients with PTC had smaller tumors than patients with PTC + PT, suggests the importance of IL-10 in HT.

Although widely considered as an anti-inflammatory cytokine, IL-10 could also induce the cytotoxicity of CD8^+^ T cells in an immune response to cancer (11). Mumm et al (2011) demonstrated that transgenic overexpression of IL-10 protected mice from carcinogenesis (20). The basis for CD8^+^ T-cell cytotoxicity in cancer is the recognition of MHC class I molecules, which carry a cancer-derived peptide. However, loss of MHC class I expression is a frequently encountered mechanism of immune escape in malignant diseases (13, 14). The restoration of MHC class I expression, on the other hand, could potentiate tumor immunity (13, 21). Downregulation of MHC class I in both PTC tumor and adjacent paratumor tissue was clearly shown on the IHC staining pictures. Results in our study also revealed significantly higher expression of MHC class I in patients with PTC + HT compared with patients with PTC, with the HLA-A gene serving as the major contributor.

Our previous results demonstrated that concomitant HT resulted in higher MHC class I expression in PTC. Moreover, administration of IL-10 effectively induced MHC class I expression on PTC cell lines K1 and TPC-1. Coculture systems containing IL-10-pretreated PTC cells and CD3^+^CD8^+^CD25^+^ T cells were employed to further examine the possible impact of IL-10 on tumor immunity. T cells cocultured with IL-10-stimulated cells of K1 and TPC-1 exhibited increased cell activation (%CD25^+^ of CD3^+^ T cells) and IL-2 production at an E:T ratio of 30:1, which was evidence for promotion in tumor immunity in PTC. The insignificant results in cocultures at an E:T ratio of 10:1 emphasized the importance of the amount of CD8^+^ T cells. Naturally, with more CD8^+^ T cells comes greater cytotoxicity. In contrast to IL-2, the concentration of IFN-γ in IL-10-stimulated cocultures dropped dramatically compared with untreated groups. IFN-γ secreted by activated T cells is essentially regarded as an antitumor cytokine and a key factor to the induction of cytotoxic T lymphocytes. Nevertheless, evidence demonstrating IFN-γ-facilitated carcinogenesis of colorectal carcinoma and melanoma indicates the opposite effect of this molecule (22, 23). Our result was another proof for the association of IFN-γ and immune escape, which may lead to tumor progression. The PD-1 expression on activated T cells cocultured with IL-10-pretreated K1 cells was downregulated significantly, revealing a suppressed T-cell apoptosis as expected.

As the other half of the co-stimulatory molecules, PD-L1 expression was also investigated. Tissue samples showed that HT reduced PD-L1 expression in patients with PTC, which may consequently promote T-cell cytotoxicity. Consistent with the coculture results, IL-10 stimulation successfully induced downregulation of PD-L1 expression in PTC cells. IFN-γ was previously reported to induce PD-L1 expression in ovarian cancer (24). Based on our results, IFN-γ may also serve as a barrier to tumor immunity in PTC.

The current study has some limitations that could compromise our findings. The sample number is relatively small, which warrants further investigations with expanded patient cohorts for this topic. Moreover, we were unable to analyze the recurrence between patients with PTC with or without HT and patients with a high IL-10 or low IL-10 expression due to insufficient survival data. The result should be interpreted with caution because cytokine IL-10 is not an equivalent for HT. We discovered an induced expression of IL-10 by HT and further revealed an enhanced tumor immunity associated with elevated IL-10. Whether or not this phenomenon could improve PTC prognosis has not been proved. Also, the relationship between IL-10-induced MHC class I expression and the PD-1/PD-L1 pathway needs further confirmation.

In conclusion, IL-10 successfully recovers MHC class I expression and enhances tumor antigenicity in PTC with concomitant HT. This study may provide new insights for cancer immunotherapy.

## Abbreviations

CD: cluster of differentiation
ELISA: enzyme-linked immunosorbent assay
E:T: effector cell:targeted cell
HLA: human leukocyte antigen
HT: Hashimoto thyroiditis
IHC: immunohistochemistry
IFN: interferon
IL: interleukin
MHC: major histocompatibility complex
PBL: peripheral blood lymphocyte
PD-1: programmed death-1
PD-L1: programmed death-ligand 1
PT: paratumor
PTC: papillary thyroid cancer
T: tumorous
TC: thyroid cancer
TNM: tumor, node, metastasis

## Additional Information

***Disclosure Summary:*** The authors declare that they have no conflict of interest.

***Data Availability:*** All data generated or analyzed during this study are included in this published article or in the data repositories listed in References.
